# 2-Pyridinecarboxaldehyde-Modified Chitosan–Silver Complexes: Optimized Preparation, Characterization, and Antibacterial Activity

**DOI:** 10.3390/molecules28196777

**Published:** 2023-09-23

**Authors:** Zhaoyu Zhang, Yurong Zhao, Zhang Hu, Zhenyu Si, Ziming Yang

**Affiliations:** 1 Faculty of Chemistry and Environmental Science, Guangdong Ocean University, Zhanjiang 524088, China; m18238796259@163.com (Z.Z.); zhaoyurong031@163.com (Y.Z.); a11931222@163.com (Z.S.); 2 South Subtropical Crop Research Institute, Chinese Academy of Tropical Agricultural Sciences, Zhanjiang 524001, China; yangziming2004@163.com

**Keywords:** modified chitosan, silver, complex, response surface methodology, antibacterial

## Abstract

The widespread prevalence of infectious bacteria is one of the greatest threats to public health, and consequently, there is an urgent need for efficient and broad-spectrum antibacterial materials that are antibiotic-free. In this study, 2-pyridinecarboxaldehyde (PCA) was grafted onto chitosan (CS) and the modified CS coordinated with silver ions to prepare PCA-CS-Ag complexes with antibacterial activity. To obtain complexes with a high silver content, the preparation process was optimized using single-factor experiments and response surface methodology. Under the optimal preparation conditions (an additional amount of silver nitrate (58 mg), a solution pH of 3.9, and a reaction temperature of 69 °C), the silver content of the PCA-CS-Ag complex reached 13.27 mg/g. The structure of the PCA-CS-Ag complex was subsequently verified using ultraviolet–visible spectroscopy, Fourier-transform infrared spectroscopy, proton nuclear magnetic resonance spectroscopy, and thermogravimetric analysis. Furthermore, three possible complexation modes of the PCA-CS-Ag complex were proposed using molecular mechanics calculations. The results of the antibacterial assay in vitro showed that the PCA-CS-Ag complex exhibited strong antibacterial activity against both Gram-positive and Gram-negative bacteria, exerting the synergistic antibacterial effect of modified chitosan and silver ions. Therefore, the PCA-CS-Ag complex is expected to be developed as an effective antibacterial material with promising applications in food films, packaging, medical dressings, and other fields.

## 1. Introduction

The widespread prevalence of infectious bacteria is one of the greatest threats to public health [1]. The invention and use of antimicrobial agents and antibiotics have lowered the morbidity and lethality of infectious diseases caused by microorganisms in humans. Unfortunately, many broad-spectrum antibiotics clinically used to prevent and control infections have been misused over time, resulting in the emergence of drug-resistant pathogens [2]. Therefore, antibiotic-free, broad-spectrum, and highly efficient antibacterial materials are urgently required.

It has long been well known that silver (Ag) has powerful antibacterial properties. Because it has non-specific bactericidal effects on broad-spectrum bacteria and is not susceptible to inducing bacterial resistance, silver has been widely used as an antimicrobial agent for wound infections and skin burns [3]. In clinical practice, silver ions are widely used in the form of silver sulfadiazine products. However, this product suffers from some drawbacks, such as poor skin permeability and possible allergic reactions to sulfadiazine components [4]. To overcome these shortcomings and fully utilize the benefits of silver, the development of safe and efficient silver-containing antimicrobial agents has been promoted [5]. Among them, silver complexes have been extensively studied, and most of them exhibited stronger antimicrobial activities than the precursor ligands, indicating the importance of silver chelation. Six novel Ag-N-heterocyclic carbene (NHC) complexes were synthesized under an argon atmosphere using standard Schlenk techniques and showed good antibacterial activity against two selected bacterial strains [6]. Chakraborty et al. developed an Ag(I) complex derived from 2,6-bis(benzothiazole)-pyridine as a ligand, and its zone of inhibition in soft tissue and skin infection models was comparable to that of silver nitrate, exerting strong antibacterial effects on Gram-positive and Gram-negative bacterial strains [7].

In particular, natural polymer-based silver complexes have attracted an increasing amount of research attention in recent years due to their unique physicochemical and biological properties. Ibrahim et al. synthesized chitosan–silver complexes by using chitosan as a neutral ligand and applied the complexes as biomedical devices. In animal defect models, the chitosan–silver complex treated group (1.2 mg per mm^2^ of bone defect) showed faster and well-organized bone formation in comparison to the control group, demonstrating that the chitosan–silver complex displayed a significant potential bone regenerator in rat mandibles [8]. Leone et al. developed a phosphorylated xanthan gum–Ag(I) complex (XGP–Ag) by virtue of the strict coordination ability of polymers with silver ions, and the diluted solution of the XGP–Ag complex (XGP–Ag 0.02%) showed no cytotoxicity and had an excellent capability to inhibit bacterial strain (*Pseudomans fluorescens* and *Staphylococcus epidermidis*) proliferation, of about 50%, showing a comparable behavior to in comparison to phosphorylated polymer and about 30% in comparison with pure tryptic soy broth [9].

Chitosan (CS), a deacetylated product of chitin, is currently the only positively charged natural biopolymer found in nature. Due to its superior properties, such as film-forming, non-toxicity, biocompatibility, and biodegradability, CS has been widely used in many fields including food, medicine, and tissue engineering [10,11]. As the main representative of natural antibacterial agents, CS has broad-spectrum antibacterial properties, but its antibacterial ability is insufficient and limited by many factors [12]. Meanwhile, due to the rigid crystalline structure of unmodified CS, it has some drawbacks such as poor solubility in organic solvents and water, low drug-loading capacity, high viscosity, and pH sensitivity, which limit its applications [13,14]. CS contains a large number of amino and hydroxyl groups, which allow the introduction of other functional groups into the molecular chain to improve its functionality and increase its utilization value. Recently, extensive studies have been devoted to the development of CS derivatives and the application of their antibacterial properties, with some research progress being achieved [15,16,17].

Due to the preparation of natural polymer–silver complexes triggering the application exploration of new antibacterial materials, there is a strong demand for the fabrication of natural polymer-silver complexes. Herein, the present study was designed to introduce a heterocyclic bidentate structure onto chitosan to increase the coordination sites of CS available to chelate silver ions. By optimizing the fabrication process, the PCA-CS-Ag complex that can chelate silver ions with high efficiency was obtained, which not only effectively overcame the disadvantage of the easy migration of silver ions, but also enhanced the antibacterial activity due to the synergistic effect of the modified chitosan and silver ions. In addition, the prepared PCA-CS-Ag complex was structurally characterized and its antibacterial activity was evaluated.

## 2. Results and Discussion

### 2.1. Preparation of PCA-CS-Ag Complex

**Figure 1:**
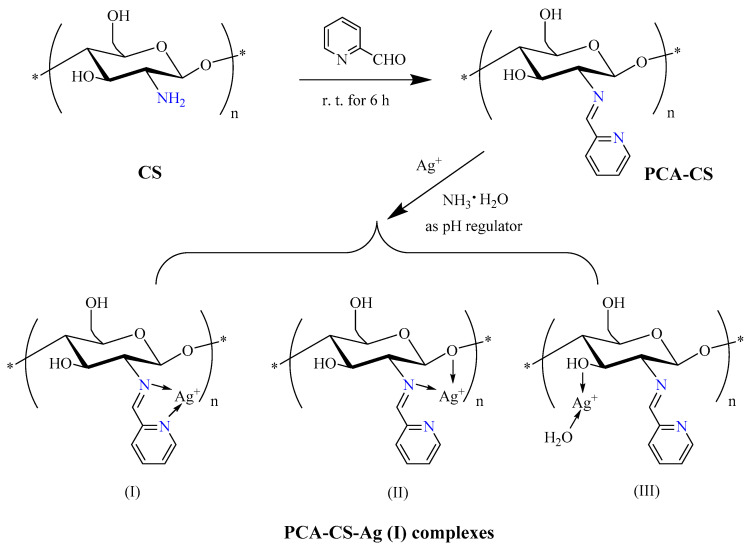
Preparation of PCA-CS-Ag complex. The symbol * represents the polymerization site of the polymer, and (I), (II), and (III) are for the chemical structures of the three coordination modes, respectively.

As shown in Figure 1, 2-pyridinecarboxaldehyde (PCA) was added into the CS solution and stirred at room temperature (r.t.) for 6 h to obtain the modified CS (PCA-CS) through the nucleophilic addition–elimination reaction. Subsequently, ammonia water was chosen as a pH regulator instead of a NaOH aqueous solution, which avoided the direct interaction between Ag^+^ and alkali to form Ag_2_O, and then PCA-CS coordinated with silver ions under the right conditions to yield the PCA-CS-Ag complexes.

### 2.2. Single-Factor Experiment Results and Analysis

#### 2.2.1. Effect of the Additional Amount of Silver Nitrate on the Silver Content

**Figure 2:**
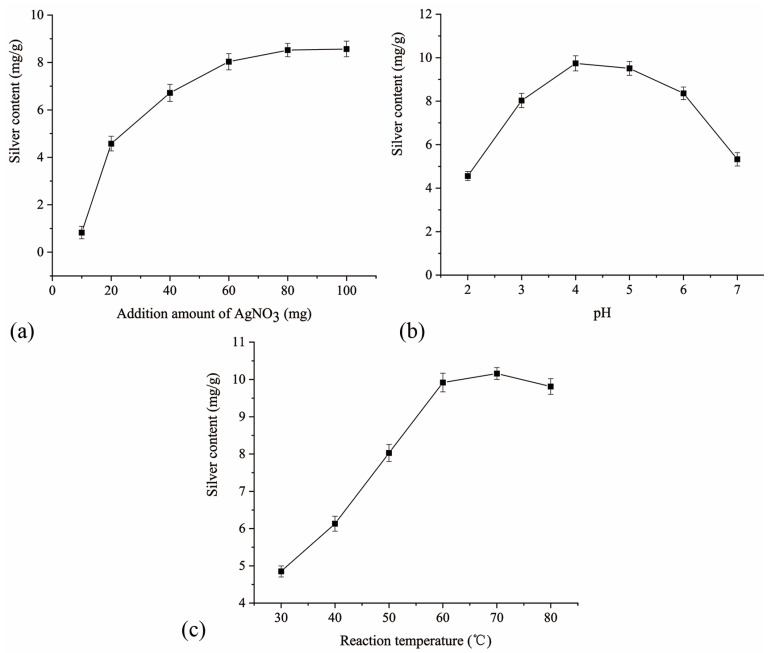
Effect of (**a**) additional amount of silver nitrate, (**b**) solution pH, and (**c**) reaction temperature on the silver content.

As shown in Figure 2a, the additional amount of silver nitrate had a significant effect on the silver content of the complex. With the increase of the additional amount of silver nitrate from 10 to 60 mg, the silver content in the resulting complex rapidly increased. However, further increasing the additional amount of silver nitrate from 60 to 100 mg, the silver content slowly increased and gradually stabilized. It should be supposed that the lone pair electrons provided by O and N in the ligand of modified CS entered the empty orbitals of silver ions due to the electrostatic effect, forming a complex. Consequently, the amount of coordination increased upon increasing the silver ion concentration. However, as the number of chelating sites in the polysaccharide molecules gradually decreased, the coordination amount increased slowly until the coordination saturated and entered a dynamic equilibrium [18]. In addition, not all of the silver ions were involved in coordination according to the mass of metal ions before and after the reaction [19].

#### 2.2.2. Effect of the Solution pH on the Silver Content

As shown in Figure 2b, the silver content in the complex increased and then decreased upon increasing the solution pH, reaching a maximum at pH = 4. The results indicated that the solution pH played an important role in the complex preparation process, which was consistent with the results of other studies [20,21]. This could be explained by the fact that since modified CS mainly coordinated with Ag^+^ via N-containing residues, when the solution pH was low, some of the N-containing residues were protonated and existed in the form of cations, which weakened the coordination capacity of the modified CS with Ag^+^ due to the electrostatic repulsion effect, reducing the amount of coordination. Upon increasing the solution pH, the number of N-containing residues with lone pair electrons increased, enhancing the coordination capability with Ag^+^, and leading to a gradual increase in the amount of coordination. However, when the solution pH exceeded 4.0, some of the silver ions were hydrolyzed and decomposed to form a precipitate, and the solubility of CS was decreased moderately, resulting in a gradual decrease in the amount of coordination.

#### 2.2.3. Effect of the Reaction Temperature on the Silver Content

From Figure 2c, as the temperature increased, the silver content in the complex first increased and then decreased, reaching a maximum (10.16 mg/g) at 70 °C. It could be speculated that the temperature affected the collision frequency between the molecules and when the temperature was too high, the movement of the polysaccharide molecules and silver ions was excessively fast, resulting in a higher dissociation rate than the binding rate. Moreover, excessively high temperatures can lead to changes in the spatial structure of polysaccharides, which may adversely affect their chelation with metal ions [22].

### 2.3. Optimization Preparation of PCA-CS-Ag Complex

#### 2.3.1. Experimental Design and Results

Based on the results of single-factor experiments, a three-factor three-level Box-Behnken experimental design was applied with the additional amount of silver nitrate (A), solution pH (B), and reaction temperature (C) as independent variables. In total, 17 experimental protocols were included and the results are shown in Table 1.

#### 2.3.2. Regression Modeling and Analysis of Variance (ANOVA)

According to the results presented in Table 1, a three-factor regression equation was established as follows:

Y(Silver content) = 13.31 − 0.31 × A − 0.21 × B − 0.34 × C − 1.12 × AB + 0.13 × AC − 0.24 × BC − 1.37 × A^2^ − 0.72×B^2^ − 2.92 × C^2^ (1)

According to ANOVA (Table 2), when using this regression equation to describe the relationship between each factor and the response value, a significant linear relationship was identified between the dependent variable and all of the independent variables. The coefficient of determination (R^2^) of the regression model was 0.9929 with *p* < 0.0001, which indicated that the regression model was highly significant. The out-of-fit term of the equation was not significant (*p* = 0.4672), indicating the good fit of the model. The adjusted R^2^ value was 0.9838, suggesting that 98.38% of the response value originated from the selected variable with very little experimental error. Therefore, this equation could replace real test points for analysis to predict the response value (silver content in the complex). The degree of influence of each factor on the response value followed the sequence of reaction temperature (*p* = 0.0057 **) > additional amount of silver nitrate (*p* = 0.0077 **) > solution pH (*p* = 0.0451 *).

**Figure 3:**
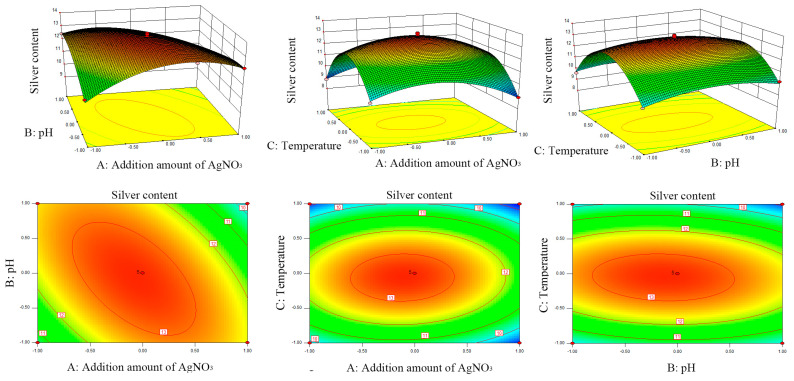
Three-dimensional response surfaces and contour plots of the dependent variable (silver content) and three independent variables (additional amount of silver nitrate, solution pH, and reaction temperature).

Response surface methodology overcomes the disadvantage of orthogonal experiments that cannot provide visual images, producing three-dimensional response surfaces and contour plots for the interaction between the three experimental factors based on a quadratic equation. The steeper the surface and the closer to the ellipse of the contour line, the more significant the influence [23]. As shown in Figure 3, the response surface of the additional amount of silver nitrate (A) and solution pH (B) was steep with an ellipse-shaped contour plot, which indicated that the AB interaction had a highly significant influence on the response value (*p* < 0.001).

#### 2.3.3. Optimization Validation

According to the software optimization, the optimal preparation parameters were an additional amount of silver nitrate (58.2 mg), a solution pH of 3.94, and a reaction temperature of 69.4 °C. The predicted silver content in the complex was 13.34 mg/g. Validation experiments were carried out to test the reliability of the optimized results. Considering the actual operation process, the optimized parameters were adjusted to an additional amount of silver nitrate (58 mg), solution pH of 3.9, and reaction temperature of 69 °C. Three parallel validation tests were performed and the silver content in the complex was 13.27 mg/g with a relative error of 0.52%. The complex prepared via the optimal process was selected for subsequent structural characterization and performance studies.

### 2.4. UV–Vis Analysis

**Figure 4:**
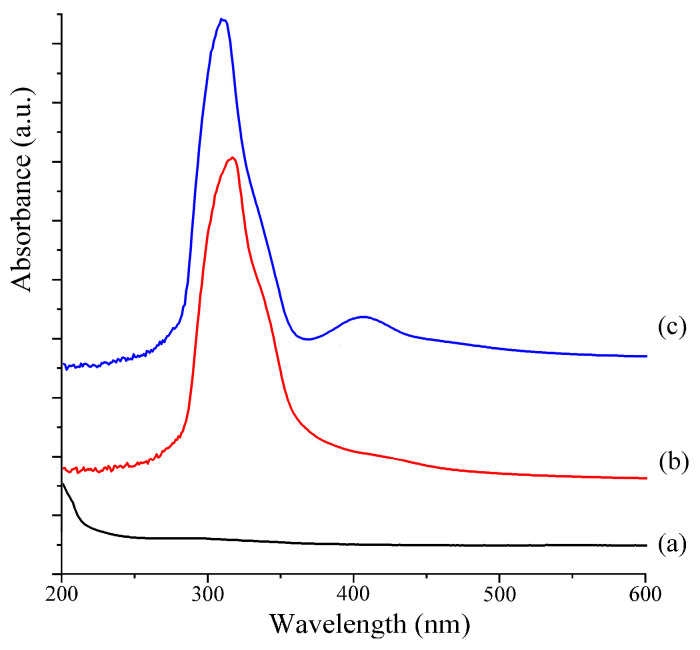
UV–Vis spectra of CS (**a**), PCA-CS (**b**), and PCA-CS-Ag complex (**c**).

UV–Vis spectroscopy is a method of analyzing compounds based on their varying ultraviolet absorption intensities at different wavelengths of light, which is one of the simplest and most affordable methods. The UV–Vis spectra obtained for CS, PCA-CS, and PCA-CS-Ag are presented in Figure 4. Due to its high degree of deacetylation (≥95%), CS contains few n-π* electronic transition groups (CH_3_CO-) in its molecular structure. Therefore, no obvious absorption peak was observed for CS in the wavelength range studied. When compared with CS, PCA-CS exhibited a strong absorption peak at 317 nm, which was attributed to the K band generated by the π-π* electronic transition of the conjugated double bonds. This indicated that a conjugated group was introduced in the molecular structure of CS, which also confirmed the formation of PCA-CS with an imine structure. After coordination with Ag^+^, the maximum absorption peak shifted from 317 to 311 nm, accompanied by a new absorption peak at 415 nm. This could be attributed to the change in the electronic spectrum caused by charge transfer in the complex, which was in agreement with previous reports [24]. The results indicated that coordination with Ag^+^ affected the molecular structure of PCA-CS, demonstrating the successful coordination between Ag^+^ and PCA-CS.

### 2.5. FT-IR Analysis

**Figure 5:**
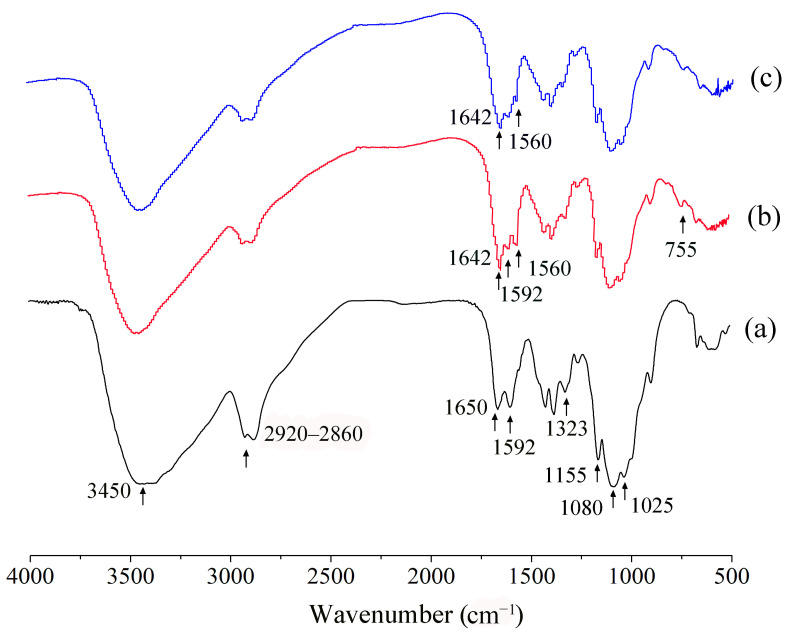
FT-IR spectra of CS (**a**), PCA-CS (**b**), and PCA-CS-Ag complex (**c**).

Infrared spectroscopy is a sensitive method used to analyze the molecular chemical structure of materials, which is suitable for determining macromolecules in different states, concentrations, and environments. Therefore, it is an effective tool for determining the changes in the chemical structure of polymers [25,26]. FT-IR spectra of CS, PCA-CS, and the PCA-CS-Ag complex are shown in Figure 5. In the FT-IR spectrum obtained for CS, the absorption peak at 3450 cm^−1^ was attributed to the superposition of the N-H and O-H stretching vibrations, and the absorption band at 2920–2860 cm^−1^ was caused by the symmetric and asymmetric stretching vibrations of C-H. The absorption peaks at 1650, 1592, and 1323 cm^−1^ were assigned to the stretching vibrations of the carbonyl group (C=O), the bending vibrations of the amino group (N-H), and the stretching vibrations of the carbon–nitrogen bond (C-N), respectively, corresponding to the classic Amide I, Amide II, and Amide III bands of CS [27]. In addition, the absorption peaks at 1155, 1080, and 1025 cm^−1^ were attributed to the stretching vibrations of C-O-C, secondary, and primary alcohols on the pyran ring of CS, respectively [28]. When compared with CS, the amino group (N-H) bending vibration peak observed at 1592 cm^−1^ in the spectrum of PCA-CS was weakened and new absorption peaks appeared at 1642, 1560, and 755 cm^−1^, which were assigned to the stretching vibrations of the imine (C=N) bonds, the stretching vibrations of the pyridine C=C bonds, and the bending vibrations of the pyridine C=C–H bonds, respectively [29], indicating that PCA was successfully grafted on CS. When compared with PCA-CS, the absorption peaks at 1642 and 1560 cm^−1^ were weakened for the PCA-CS-Ag complex, indicating the coordination of Ag^+^ with the imine moieties and pyridine ring, which was in agreement with previous studies in the literature [30]. Meanwhile, the absorption peaks at 1080 and 1025 cm^−1^ did not change obviously, which suggested that only a small amount of the hydroxyl groups may be coordinated with Ag^+^.

### 2.6. ^1^H NMR Analysis

**Figure 6:**
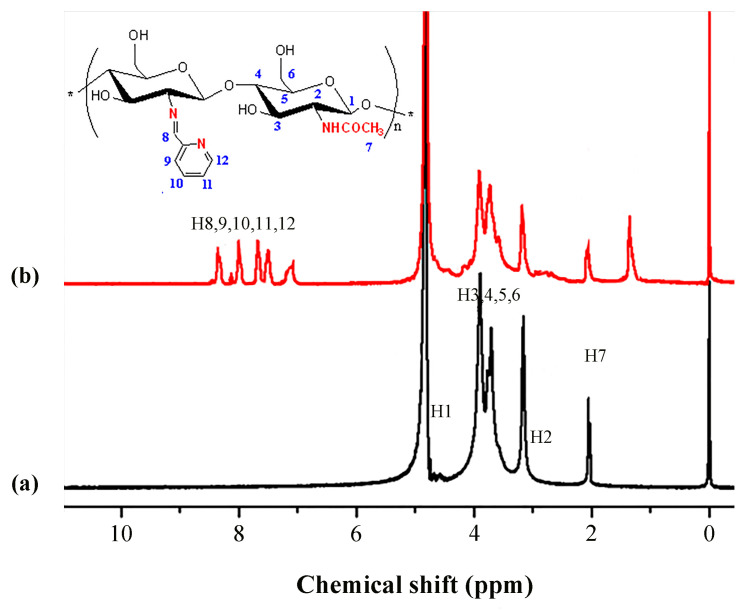
^1^H NMR spectra of CS (**a**) and PCA-CS (**b**). The symbol * represents the polymerization site of the polymer.

To further confirm that PCA was successfully grafted onto CS, a structural analysis was carried out using ^1^H NMR spectroscopy. The ^1^H NMR spectra obtained for CS and PCA-CS are shown in Figure 6. In the CS spectrum, the peak at 2.02 ppm was attributed to the CH_3_ protons in the N-acetylamino residues. The peak corresponding to the H1 proton was located near 5.00 ppm, which was covered by the solvent peak and could not be recognized. The signal peak located at 3.17 ppm suggested the presence of the H2 protons and the response signals of H3–H6 were in the range of 3.32–3.95 ppm where overlapping peaks were observed, which was consistent with those reported in the literature [31,32]. When compared with CS, new signals in the range of 7.0–8.5 ppm were observed in the spectrum obtained for PCA-CS. The signal at 8.08 ppm corresponded to the imine protons, while those at 8.45, 7.83, 7.67, and 7.24 ppm corresponded to the H12, H9, H10, and H11 protons on the pyridine ring residues, respectively [33,34]. These results further confirmed the successful grafting of PCA onto CS.

### 2.7. TG Analysis

**Figure 7:**
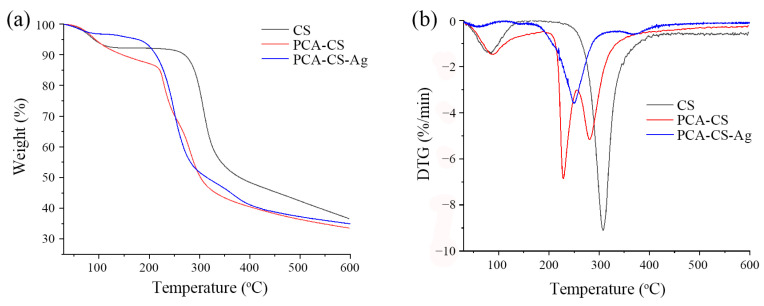
TG (**a**) and DTG (**b**) curves of CS, PCS-CS, and PCA-CS-Ag complex.

According to the TG curves shown in Figure 7a, the weight losses of pure CS, PCA-CS, and PCA-CS-Ag in the first stage (30–120 °C) were 7.21%, 8.77%, and 3.37%, respectively. The weight loss in this stage was caused by the loss of bound and free water in the samples [35]. These results suggested that the water content in PCA-CS was higher than those of CS and PCA-CS-Ag, indicating that grafting PCA enhanced the water absorption ability of CS and subsequent coordination with Ag^+^ reduced the water content, which was consistent with previous studies [36]. The second stage (120–400 °C) was attributed to the thermal degradation of the CS chains. The onset degradation temperatures of CS, PCA-CS, and PCA-CS-Ag were 258, 215, and 193 °C, respectively, which indicated that the thermal stability of CS decreased after chemical modification and was further weakened upon coordination with Ag^+^, similar to the thermal stability observed for Schiff base–Zn complexes [37]. At the end of the thermal degradation process (600 °C), the remaining mass ratios of CS, PCA-CS, and PCA-CS-Ag were 36.43%, 33.49%, and 34.87%, respectively. The higher residual amount of PCA-CS-Ag than that of PCA-CS further confirmed the coordination between PCA-CS and Ag^+^ [38].

According to the DTG curves (Figure 7b), the dehydration peaks of CS, PCA-CS, and PCA-CS-Ag were located at 83, 88, and 62 °C, respectively, indicating that PCA-CS had a strong ability to bind water and, thus, required a higher amount of energy to vaporize the water molecules compared with CS. In the subsequent decomposition of the polymer, the weight loss peaks of CS and PCA-CS-Ag were located at 308 and 250 °C, respectively, while two weight loss peaks at 228 and 281 °C were observed for PCA-CS. The two peaks observed for PCA-CS may correspond to the thermal decomposition of the grafted Schiff base residues and pyranose rings of CS, respectively. Taken together, the chemical modification using PCA and coordination with Ag^+^ reduced the thermal stability of CS, which might be due to chemical grafting and coordination destroying the crystalline structure of CS, thus, resulting in low thermal tolerance.

### 2.8. Molecular Computation Study

**Figure 8:**
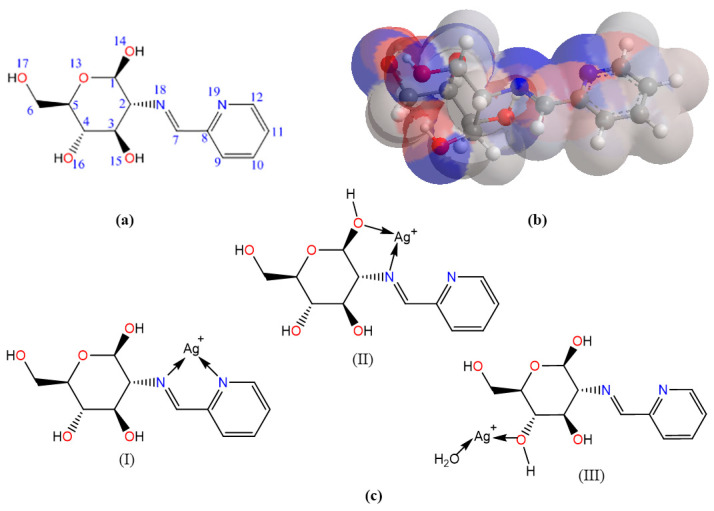
The optimized geometrical structure of the PCA-CS monomer (**a**), colored atom electron cloud distribution of the PCA-CS monomer (**b**), and the proposed coordination modes of the PCA-CS-Ag complex (**c**), and (I), (II), and (III) are for the chemical structures of the three coordination modes, respectively.

To investigate the coordination mode of PCA-CS with Ag^+^, molecular mechanics (MM2) calculations were used to optimize the molecular conformations and calculate the molecular energy and surface charge density, which will help to speculate on the formation process of polymer–metal complexes [39]. To simplify the calculations, the PCA-CS monomer was used as the research object, and its geometric structure was drawn using Chem3D software 2020 v20.0.0.41 (Figure 8a). The total potential energy of a molecule is the algebraic sum of the following energy terms, including stretching (stretch), bending (bend), stretch-bending (stretch-bend), torsion deformation (torsion), van der Waals force (VDW), and dipole-dipole force (Dipole/Dipole) [40]. The total energy of the optimized molecular conformation of the PCA-CS monomer was 26.6016 kcal/mol.

Based on the optimized conformation of the PCA-CS monomer, the atomic charges of the molecule were calculated using the Hückel method to obtain the net charge distribution of the individual atoms. The atomic electron cloud density of the PCA-CS monomer conformation was colored on the surface with red and blue, representing the positive and negative charges, respectively, and the color shade represented the intensity of the charge density. As shown in Figure 8b, both oxygen and nitrogen atoms in the PCA-CS monomer carried a large number of negative charges and had strong nucleophilic abilities, indicating that they may participate in the coordination as electron donors when reacting with metal ions. There were usually two kinds of structures of chitosan–metal complexes [38,41]. One was that metal ions coordinated with hydroxyl or amino groups on a single chitosan chain to form a hanging structure. The other was that metal ions coordinated with hydroxyl or amino groups on two or more chitosan chains to form a bridge structure in a way similar to the crosslinking agent. Considering the electron cloud densities, ligand field strengths, and spatial distances, three possible coordination modes of the PCA-CS-Ag complex were proposed (Figure 8c). According to MM2 calculations (Table 3), three structures (Mode I, II, and III) had relatively low total energies of 120.8853, 125.3698, and 121.8691 kcal/mol, respectively. Therefore, it could be found that not only the nitrogen heterocyclic Schiff base participated in the coordination reaction, but also a portion of the -OH groups on the polysaccharide chain was involved, thereby increasing the number of silver ions bound to the polymer.

### 2.9. Antibacterial Activity

**Figure 9:**
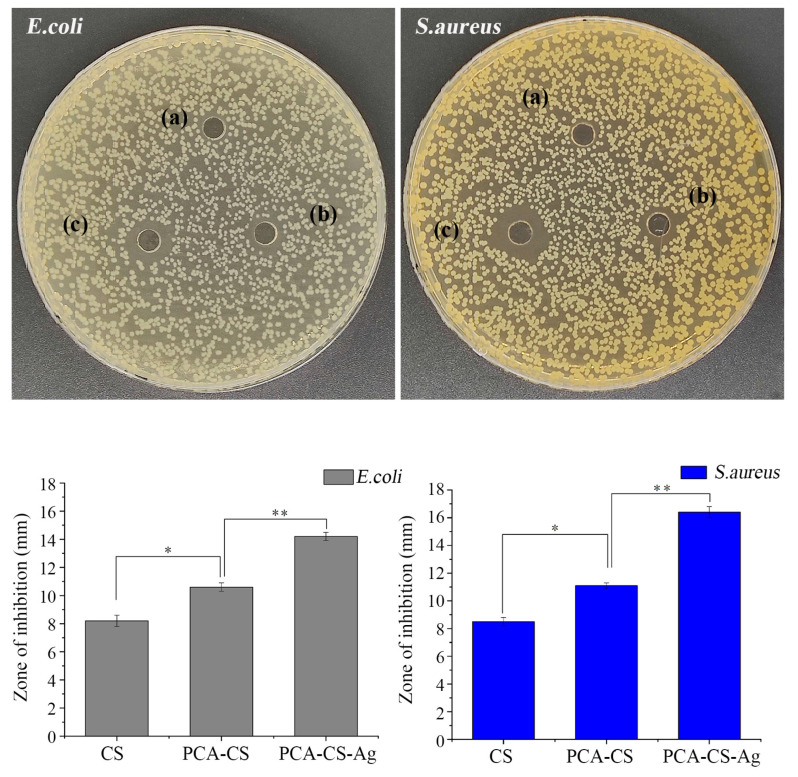
Photographs of inhibition zones ((a): CS; (b): PCA-CS; and (c): PCA-CS-Ag) and the measured results for the ZOI values, * *p* < 0.05 and ** *p* < 0.01.

Metals, such as silver and zinc, with antimicrobial activity offer a wide spectrum of applications as additives or components for biopolymers, medicine, textiles, coatings, and interior paints. Chen et al. [42] prepared the single-ion-exchange zeolite (silver zeolite, zinc zeolite) and investigated their antibacterial effects on *Escherichia coli* by measuring the O.D. value of the bacteria solution after contacting the antibacterial agent. It was found that the antibacterial effect of silver zeolites on *E. coli* with a low silver content was much better than zinc zeolites with a high zinc content, indicating silver-containing materials with better antibacterial activity compared with zinc-containing ones. Herein, the inhibition zone results obtained for the silver-containing samples are shown in Figure 9. The antibacterial effect of pure CS was not obvious, which was due to the fact that the antibacterial activity of pure CS was affected by lots of factors, such as the bacterial species, environmental factors, and its molecular structure (including biological sources, molecular weight, degree of deacetylation, structural configuration, and so on) [43]. The ZOI values of PCA-CS against *E. coli* and *Staphylococcus aureus* were 10.6 ± 0.3 and 11.1 ± 0.2 mm, respectively, presenting a significantly stronger antibacterial activity than those of CS (*p* < 0.05), which was in line with the previously reported results [29,44]. It could be explained that CS Schiff base with imine groups (-RC=N-) improved its permeability, hydrophilicity, and solubility, as well as enhanced its ability to carry positive charges and coordinate with metals, thereby contributing to its superior antibacterial efficacy. After the chelation of PCA-CS with Ag^+^, the ZOI values of PCA-CS-Ag against *E. coli* and *S. aureus* were 14.2 ± 0.3 and 16.2 ± 0.4 mm, respectively, compared to PCA-CS with significant differences (*p* < 0.01), indicating that the coordination with Ag^+^ exerted a synergistic antibacterial effect.

**Figure 10:**
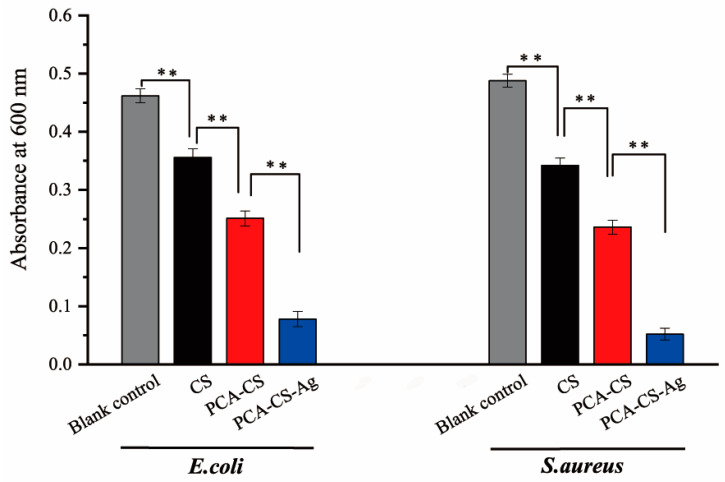
Absorbance of bacteria suspension at 600 nm after co-culture with the samples, ** *p* < 0.01.

To further confirm their antibacterial activities, the samples were co-cultured with bacterial suspensions for 5 h, and the optical density (OD) value of the bacterial suspensions was measured at 600 nm to quantitatively evaluate the antibacterial effects. As shown in Figure 10, CS in the bacterial suspensions showed antibacterial effects with no more than 30% of antibacterial rates. The chemical modification and the complexation with Ag^+^ significantly enhanced the antibacterial effect of the polymer, which was consistent with the results of ZOI. Especially, PCA-CS-Ag showed excellent antibacterial activity against *E. coli* and *S. aureus* with antibacterial rates of 83.1% and 89.3%, respectively (Table 4).

The antibacterial effect of the PCA-CS-Ag complex can be explained using three pathways. (I) CS antibacterial pathway; the antibacterial mechanism of CS was complex and mainly manifested in the following aspects: (1) CS with polycationic properties interacted with the anionic cell wall components of bacteria, leading to the externalization of the intracellular components [45]; (2) due to its good film-forming properties, CS formed a polymeric film on the surface of bacterial cells, which directly blocked the input of nutrients and oxygen [46]; (3) CS inhibited the synthesis of mRNA and protein by penetrating into the nuclei of microbial cells [47]; and (4) the chelation of CS with metals prevented the growth of microorganisms by inhibiting spores and their binding to essential nutrients [48]. In particular, PCA-CS with increased permeability, hydrophilicity, and solubility had a stronger ability to carry positive charges. Meanwhile, the imine groups (-N=C-) of PCA-CS provided excellent metal-binding properties and, thus, inhibited the growth of bacteria by interacting with the cellular components [44,49]. (II) Silver antibacterial pathway; Ag^+^ had a high affinity for compounds containing S and P, and was bound to proteins in the cell wall of bacteria, inactivating them, and generating reactive oxygen species, which further disrupted the permeability of the cell membrane, leading to leakage of intracellular substances [50,51,52]. In addition, Ag^+^ bound to the DNA of bacteria, damaged the DNA, and thus, inhibited the growth and reproduction of bacteria [53]. (III) Complex antibacterial pathway; due to the hydrophilic and cationic properties of PCA-CS-Ag, microorganisms tended to adhere to the polymer chains of PCA-CS-Ag, and the released Ag^+^ rapidly interacted with the microbial proteins and DNA, resulting in an excellent antimicrobial performance. In summary, the antibacterial mechanism of the PCA-CS-Ag complex may be attributed to the synergistic antibacterial effects of PCA-CS and silver ions.

## 3. Materials and Methods

### 3.1. Materials

Chitosan (deacetylation degree ≥ 95%, viscosity of 100–200 mPa.s), 2-pyridinecarboxaldehyde (AR, 98%), silver nitrate (AR, 99.8%), and glacial acetic acid (AR, 99.5%) were purchased from Shanghai Aladdin Bio-Chem Technology Co., Ltd., China. Escherichia coli (*E. coli*, ATCC 25922) and Staphylococcus aureus (*S. aureus*, ATCC 29213) were gained from Guangdong Microbial Culture Collection Center, Guangzhou, China. All other reagents and chemicals were of analytical grade and supplied by Zhanjiang Kecheng Trading Co., Ltd., Zhanjiang, China.

### 3.2. Synthesis of 2-Pyridinecarboxaldehyde-Modified Chitosan (PCA-CS)

CS (0.5 g) was added to the glacial acetic acid solution (50 mL, 1%, *v*/*v*) and dissolved under stirring to form a transparent solution. PCA (2 equiv. glucosamine unit of CS) was then added and the resulting mixture was stirred for 6 h at room temperature. Subsequently, a solution of NaOH (20 mL, 3%, *w*/*v*) was added slowly to the reaction mixture for precipitation. The mixture was then subjected to filtration, washed successively with distilled water and 95% ethanol, and vacuum-dried to obtain PCA-CS.

### 3.3. Preparation of PCA-CS-Ag Complex

The prepared PCA-CS (0.5 g) was dissolved in glacial acetic acid solution (50 mL, 1%, *v*/*v*) and the required amount of silver nitrate solution (50 mmol/L) was added using a constant pressure dropping funnel under a nitrogen atmosphere. The pH of the solution was adjusted with an ammonia solution (10%, *v*/*v*) and the resulting mixture was kept from light and stirred for 3 h at a specified temperature. Afterwards, 50 mL of acetone was added for precipitation and the resulting mixture was filtered to collect the precipitate. The precipitate was successively washed with distilled water, 95% ethanol, and anhydrous ethanol, followed by drying under vacuum to obtain the product. The silver content of the complex was analyzed using inductively coupled plasma mass spectrometry (ICP-MS).

### 3.4. Investigation of the Influence of the Preparation Conditions

By using single-factor experiments, three independent variables of the reaction conditions used for complex preparation were investigated, including the additional amount of silver nitrate (10, 20, 40, 60, 80, and 100 mg), solution pH (2, 3, 4, 5, 6, and 7), and reaction temperature (30, 40, 50, 60, 70, and 80 °C). When one variable was examined, the others were fixed at a specified value.

### 3.5. Box–Behnken Design

Based on the results of single-factor experiments, a three-factor three-level Box–Behnken design was applied using the additional amount of silver nitrate, solution pH, and reaction temperature as independent variables, and the silver content of the complex was used as the response value to optimize the preparation process.

### 3.6. Ultraviolet–Visible (UV–Vis) Spectroscopy

The test sample was dissolved in acetic acid solution (1%, *v*/*v*) and its UV–Vis spectrum was recorded on a UV–Vis spectrometer (UV-3700, Shimadzu, Kyoto, Japan) using acetic acid solution as the reference solution.

### 3.7. Fourier-Transform Infrared (FT-IR) Spectroscopy

A small amount of sample and potassium bromide (mass ratio of sample to potassium bromide = 1:100) was added to a mortar, stirred, ground, and pressed into a thin pellet. The pellet was then analyzed on an FT-IR spectrometer over the wavelength range of 400–4000 cm^−1^ over 16 scans.

### 3.8. Proton Nuclear Magnetic Resonance (^1^H NMR) Spectroscopy

The ^1^H NMR spectra of the samples were recorded on a Bruker Avance DPX-400 spectrometer (Bruker, Billerica, MA, USA), and DCl/D_2_O (1/100, *v*/*v*) was used as the solvent.

### 3.9. Thermogravimetric (TG) Analysis

An appropriate amount of sample was taken for TG analysis on a TG/DTA 6300 thermal analyzer (Perkin Elmer, Waltham, MA, USA) using nitrogen as the carrier gas at a flow rate of 50 mL/min. The TG and DTG curves were recorded over the temperature range from 30 to 600 °C at a heating rate of 10 °C/min.

### 3.10. Molecular Computation Study

Theoretical calculations on the molecular structure were carried out using Chemoffice suite 2020 v20.0.0.41 software from PerkinElmer (Waltham, MA, USA).

### 3.11. Antibacterial Activity Determination

#### 3.11.1. Zone of Inhibition (ZOI) Assay

Gram-positive *S. aureus* and Gram-negative *E. coli* were selected as the testing bacteria to determine the in vitro antibacterial activity. Briefly, a sterilized culture medium solution was poured into a petri dish under sterile conditions and allowed to solidify. The bacteria was diluted to 10^5^ CFU/mL using a sterilized PBS solution and the bacterial suspension (100 μL) was added to the solidified culture medium and spread well using a sterile swab. A hole punch was used to punch holes and the sample solution (20 μL) was added to the hole. The petri dish was then placed in a constant temperature incubator for 24 h at 37 °C. Photographs were taken and the ZOI value was measured. Each assay was performed in triplicate and the average value was reported.

#### 3.11.2. Determination of Antibacterial Rate with Optical Density Method

To further confirm the antibacterial activity of the samples, the optical density (OD) value of bacterial suspension was measured using a UV–Vis spectrophotometer. The concentration of the bacterial suspension was adjusted to 10^6^ CFU/mL, and the sterilized sample solution was added to the bacterial suspension (50 mL). The mixture was continuously shaken with 220 r/min at 37 °C for 5 h. The bacterial suspension without the sample was used for blank control. The absorbance of the bacterial suspension was measured at 600 nm to quantitatively evaluate the antibacterial effect of samples.

### 3.12. Statistical Analysis

Unless otherwise stated, the results obtained from each experiment were independently repeated three times and presented as the mean ± standard deviation. SPSS 26.0 statistical software was used to perform the statistical analysis, and the independent samples *t*-test was used to compare differences between samples; *p* < 0.05 was considered statistically significant.

## 4. Conclusions

Chitosan was chemically modified by the grafting of PCA to provide multiple chelating sites for silver ions. Based on the results of single-factor experiments, the preparation process was optimized using response surface methodology, and the PCA-CS-Ag complex with a silver content of 13.27 mg/g was prepared under the optimal preparation conditions (an additional amount of silver nitrate (58 mg), a solution pH of 3.9, and a reaction temperature of 69 °C). The PCA-CS-Ag complex was characterized using UV–Vis, FT-IR, ^1^H NMR, TG analysis, and MM2 calculations to verify its structure. The results of in vitro antibacterial experiments indicated that the PCA-CS-Ag complex possessed potent antibacterial activity against Gram-positive and Gram-negative bacteria, and exerted the synergistic antimicrobial effect of the modified CS and silver ions. In summary, this study provided notable support for the promising applications of a PCA-CS-Ag complex as a novel antibacterial agent in food materials and medical dressings.

## Figures and Tables

**Table 1:** Experiment design and results of Box−Behnken.

No.	A: Additional Amount of AgNO_3_ (mg)	B: pH	C: Temperature (°C)	Silver Content (mg/g)
1	0 (60)	−1 (3)	−1 (60)	9.92
2	0 (60)	−1 (3)	1 (80)	9.61
3	0 (60)	1 (5)	−1 (60)	10.19
4	0 (60)	1 (5)	1 (80)	8.92
5	−1 (40)	0 (4)	−1 (60)	9.64
6	−1 (40)	0 (4)	1 (80)	8.83
7	1 (80)	0 (4)	−1 (60)	8.93
8	1 (80)	0 (4)	1 (80)	8.64
9	−1 (40)	−1 (3)	0 (70)	10.81
10	−1 (40)	1 (5)	0 (70)	12.42
11	1 (80)	−1 (3)	0 (70)	12.23
12	1 (80)	1 (5)	0 (70)	9.38
13	0 (60)	0 (4)	0 (70)	13.34
14	0 (60)	0 (4)	0 (70)	12.91
15	0 (60)	0 (4)	0 (70)	13.53
16	0 (60)	0 (4)	0 (70)	13.31
17	0 (60)	0 (4)	0 (70)	13.45

**Table 2:** Analysis of variance for the regression.

Source of Variance	Sum of Square	Degree of Freedom	Mean Square	F-Value	*p*-Value ^1^
Model	57.09	9	6.34	109.22	<0.0001 **
A	0.79	1	0.79	13.67	0.0077 **
B	0.34	1	0.34	5.93	0.0451 *
C	0.90	1	0.90	15.46	0.0057 **
AB	4.97	1	4.97	85.63	<0.001
AC	0.068	1	0.068	1.16	0.3164
BC	0.23	1	0.23	3.97	0.0867
A2	7.95	1	7.95	136.87	<0.0001 **
B2	2.21	1	2.21	38.00	0.0005 **
C2	36.00	1	36.00	619.86	<0.0001 **
Residual	0.41	7	0.058	—	—
Lack of fit	0.18	3	0.059	1.03	0.4672
Pure error	0.23	4	0.057	—	—
Cor. total	57.50	16	—	—	—

^1^ * Significant, *p* < 0.05; ** Highly significant, *p* < 0.01.

**Table 3:** Energy values of molecular conformation optimization.

Energy (kcal/mol)	Monomer of PCA-CS	PCA-CS-Ag (Mode I)	PCA-CS-Ag (Mode II)	PCA-CS-Ag (Mode III)
Stretch	1.2766	2.3763	17.8308	26.6365
Bend	8.9573	51.1380	62.5756	90.8197
Stretch-Bend	0.6058	−0.0447	−0.0597	0.4352
Torsion	5.3844	59.6525	29.8432	3.9841
Non-1,4 VDW	−6.3229	−6.8230	−6.8841	−18.7602
1,4 VDW	18.3978	18.6322	21.9594	17.9010
Dipole/Dipole	−1.6975	−4.0458	0.1046	0.8527
Total Energy	26.6016	120.8853	125.3698	121.8691

**Table 4:** The antibacterial rates of CS and its derivatives.

Sample	Antibacterial Rates (%)	
	*E. coli*	*S. aureus*
CS	22.9	29.8
PCA-CS	45.7 **	51.6 **
PCA-CS-Ag	83.1 **^##^	89.3 **^##^

Compared with CS, ** *p* < 0.01; compared with PCA-CS, ^##^ *p* < 0.01.

## Data Availability

Data are contained within the article.

## References

[1] Hu F., Xia S.S., He Y., Huang Z.L., Ke H., Liao J.Z. (2022). Reactive organic radical-doped Ag(I)-based coordination compounds for highly efficient antibacterial wound therapy. Colloids Surf. B.

[2] Junaid M., Thirapanmethee K., Khuntayaporn P., Chomnawang M.T. (2023). CRISPR-based gene editing in acinetobacter baumannii to combat antimicrobial resistance. Pharmaceuticals.

[3] Lin A., Liu Y., Zhu X., Chen X., Liu J., Zhou Y., Qin X., Liu J. (2019). Bacteriaresponsive biomimetic selenium nanosystem for multidrug-resistant bacterial infection detection and inhibition. ACS Nano.

[4] Politano A.D., Campbell K.T., Rosenberger L.H., Sawyer R.G. (2013). Use of silver in the prevention and treatment of infections: Silver review. Surg. Infect..

[5] Liang X., Luan S., Yin Z., He M., He C., Yin L., Zou Y., Yuan Z., Li L., Song X. (2018). Recent advances in the medical use of silver complex. Eur. J. Med. Chem..

[6] Ulu Ö.D., Kuruçay A., Ateş B., Özdemir İ. (2023). Synthesis, characterization, in vitro antibacterial, and anticancer studies of Ag(I)-N-heterocyclic carbene (NHC) complexes. Chem. Pap..

[7] Chakraborty I., Pinto M., Stenger-Smith J., Martinez-Gonzalez J., Mascharak P.K. (2019). Synthesis, structures and antibacterial properties of Cu(II) and Ag(I) complexes derived from 2,6-bis(benzothiazole)-pyridine. Polyhedron.

[8] Ibrahim F.M., El-Hawary Y.M., Butler I.S., Mostafa S.I. (2014). Bone repair stimulation in rat mandible by new chitosan silver(I) complexes. Int. J. Polym. Mater. Polym. Biomater..

[9] Leone G., Pepi S., Consumi M., Mahdizadeh F.F., Lamponi S., Magnani A. (2021). Phosphorylated xanthan gum-Ag(I) complex as antibacterial viscosity enhancer for eye drops formulation. Carbohydr. Polym..

[10] Chen Y., Liu Y., Dong Q., Xu C., Deng S., Kang Y., Fan M., Li L. (2023). Application of functionalized chitosan in food. Int. J. Biol. Macromol..

[11] Fan P., Zeng Y., Zaldivar-Silva D., Agüero L., Wang S. (2023). Chitosan-based hemostatic hydrogels: The concept, mechanism, application, and prospects. Molecules.

[12] Riaz Rajoka M.S., Mehwish H.M., Wu Y., Zhao L., Arfat Y., Majeed K., Anwaar S. (2020). Chitin/chitosan derivatives and their interactions with microorganisms: A comprehensive review and future perspectives. Crit. Rev. Biotechnol..

[13] Panahi H.K.S., Dehhaghi M., Amiri H., Guillemin G.J., Gupta V.K., Rajaei A., Yang Y., Peng W., Pan J., Aghbashlo M. (2023). Current and emerging applications of saccharide-modified chitosan: A critical review. Biotechnol. Adv..

[14] Chen L., Tang J., Wu S., Wang S., Ren Z. (2022). Selective removal of Au(III) from wastewater by pyridine-modified chitosan. Carbohydr. Polym..

[15] Piegat A., Żywicka A., Niemczyk A., Goszczyńska A. (2020). Antibacterial activity of N,O-acylated chitosan derivative. Polymers.

[16] Chen X., Zhang H., Yang X., Zhang W., Jiang M., Wen T., Wang J., Guo R., Liu H. (2021). Preparation and application of quaternized chitosan- and AgNPs-base synergistic antibacterial hydrogel for burn wound healing. Molecules.

[17] Drozd N., Lunkov A., Shagdarova B., Il’ina A., Varlamov V. (2023). New N-methylimidazole-functionalized chitosan derivatives: Hemocompatibility and antibacterial properties. Biomimetics.

[18] Guo W.L., Shi F.F., Li L., Xu J.X., Chen M., Wu L., Hong J.L., Qian M., Bai W.D., Liu B. (2019). Preparation of a novel Grifola frondosa polysaccharide-chromium (III) complex and its hypoglycemic and hypolipidemic activities in high fat diet and streptozotocin-induced diabetic mice. Int. J. Biol. Macromol..

[19] Adewuyi S., Kareem K.T., Atayese A.O., Amolegbe S.A., Akinremi C.A. (2011). Chitosan-cobalt(II) and nickel(II) chelates as antibacterial agents. Int. J. Biol. Macromol..

[20] Chi Y., Li Y., Zhang G., Gao Y., Ye H., Gao J., Wang P. (2018). Effect of extraction techniques on properties of polysaccharides from Enteromorpha prolifera and their applicability in iron chelation. Carbohydr. Polym..

[21] Wang C., Chen Z., Pan Y., Gao X., Chen H. (2017). Anti-diabetic effects of Inonotus obliquus polysaccharides-chromium (III) complex in type 2 diabetic mice and its sub-acute toxicity evaluation in normal mice. Food Chem. Toxicol..

[22] Qiu J., Zhang H., Wang Z., Liu S., Regenstein J.M. (2016). Response surface methodology for the synthesis of an Auricularia auriculajudae polysaccharides-CDDP complex. Int. J. Biol. Macromol..

[23] Addo P.W., Sagili S.U.K.R., Bilodeau S.E., Gladu-Gallant F.A., MacKenzie D.A., Bates J., McRae G., MacPherson S., Paris M., Raghavan V. (2022). Microwave- and ultrasound-assisted extraction of Cannabinoids and Terpenes from Cannabis using response surface methodology. Molecules.

[24] Alarfaj N.A., Altamimi S.A., El-Tohamy M.F., Almahri A.M. (2019). Exploitation of localized surface plasmon resonance of silver/gold nanoparticles for the fluorescence quantification of angiotensin II receptor antagonists in their tablets and bio-samples. New J. Chem..

[25] Yang S., Zhang Q., Yang H., Shi H., Dong A., Wang L., Yu S. (2022). Progress in infrared spectroscopy as an efficient tool for predicting protein secondary structure. Int. J. Biol. Macromol..

[26] Ladan M.K., Glavač N.K. (2022). Statistical FT-IR spectroscopy for the characterization of 17 vegetable oils. Molecules.

[27] Fan L., Zou S., Ge H., Xiao Y., Wen H., Feng H., Liu M., Nie M. (2016). Preparation and characterization of hydroxypropyl chitosan modified with collagen peptide. Int. J. Biol. Macromol..

[28] Tan W., Zhang J., Mi Y., Li Q., Guo Z. (2022). Synthesis and characterization of α-lipoic acid grafted chitosan derivatives with antioxidant activity. React. Funct. Polym..

[29] Anush S.M., Vishalakshi B., Kalluraya B., Manju N. (2018). Synthesis of pyrazole-based schiff bases of chitosan: Evaluation of antimicrobial activity. Int. J. Biol. Macromol..

[30] Li P., Zhao J., Chen Y., Cheng B., Yu Z., Zhao Y., Yan X., Tong Z., Jin S. (2017). Preparation and characterization of chitosan physical hydrogels with enhanced mechanical and antibacterial properties. Carbohydr. Polym..

[31] Shen J., Nada A.A., Abou-Zeid N.Y., Hudson S.M. (2020). Synthesis of chitosan iodoacetamides via carbodiimide coupling reaction: Effect of degree of substitution on the hemostatic properties. Carbohydr. Polym..

[32] Liang S., Dang Q., Liu C., Zhang Y., Wang Y., Zhu W., Chang G., Sun H., Cha D., Fan B. (2018). Characterization and antibacterial mechanism of poly(aminoethyl) modified chitin synthesized via a facile one-step pathway. Carbohydr. Polym..

[33] Wang Z., Wang X., Zhang S., Wang Z., Gao F., Li H. (2021). Simple and prompt protonation of new dyes containing double conjugated imine bonds to strengthen the protection of copper in aggressive sulfuric acid solution. J. Mol. Liq..

[34] Rangel Rangel E., Maya E.M., Sánchez F., de la Campa J.G., Iglesias M. (2015). Palladium-heterogenized porous polyimide materials as effective and recyclable catalysts for reactions in water. Green Chem..

[35] Nina M., Fathana H., Iqhrammullah M. (2022). Preparation and characterization of new magnetic chitosan-glycine-PEGDE (Fe3O4/Ch-G-P) beads for aqueous Cd(II) removal. J. Water Process Eng..

[36] Foroughnia A., Khalaji A.D., Kolvari E., Koukabi N. (2021). Synthesis of new chitosan Schiff base and its Fe_2_O_3_ nanocomposite: Evaluation of methyl orange removal and antibacterial activity. Int. J. Biol. Macromol..

[37] Barbosa H.F.G., Cavalheiro É.T.G. (2019). The influence of reaction parameters on complexation of Zn(II) complexes with biopolymeric Schiff bases prepared from chitosan and salicylaldehyde. Int. J. Biol. Macromol..

[38] Ma F., Li P., Zhang B., Wang Z. (2017). The facile synthesis of a chitosan Cu(II) complex by solution plasma process and evaluation of their antioxidant activities. Int. J. Biol. Macromol..

[39] Mukred R.A., Yehya F.B., Al-Gabr H.M., Al Fakih M.A. (2022). New bioactive Co (II) coordination polymers with morphline and carboxylate ligands; Synthesis, structures, spectroscopic and thermal properties. J. Polym. Res..

[40] Adhikari J., Bhattarai A., Chaudhary N.K. (2022). Synthesis, characterization, physicochemical studies, and antibacterial evaluation of surfactant-based Schiff base transition metal complexes. Chem. Pap..

[41] Higazy A., Hashem M., ElShafei A., Shaker N., Hady M.A. (2010). Development of antimicrobial jute packaging using chitosan and chitosan-metal complex. Carbohydr. Polym..

[42] Chen Y., Wang L., Jiang S., Yu H.J. (2003). Study on novel antibacterial polymer materials (I) preparation of zeolite antibacterial agents and antibacterial polymer composite and their antibacterial properties. J. Polym. Mater..

[43] Ardean C., Davidescu C.M., Nemeş N.S., Negrea A., Ciopec M., Duteanu N., Negrea P., Duda-Seiman D., Musta V. (2021). Factors Influencing the Antibacterial Activity of Chitosan and Chitosan Modified by Functionalization. Int. J. Mol. Sci..

[44] Hassan M.A., Omer A.M., Abbas E., Baset W.M.A., Tamer T.M. (2018). Preparation, physicochemical characterization and antimicrobial activities of novel two phenolic chitosan Schiff base derivatives. Sci. Rep..

[45] Severino R., Ferrari G., Vu K.D., Donsì F., Salmieri S., Lacroix M. (2015). Antimicrobial effects of modified chitosan based coating containing nanoemulsion of essential oils, modified atmosphere packaging and gamma irradiation against Escherichia coli O157:H7 and Salmonella Typhimurium on green beans. Food Control.

[46] Hosseinnejad M., Jafari S.M. (2016). Evaluation of different factors affecting antimicrobial properties of chitosan. Int. J. Biol. Macromol..

[47] Tian Y., Wu D., Wu D., Cui Y., Ren G., Wang Y., Wang J., Peng C. (2022). Chitosan-Based Biomaterial Scaffolds for the Repair of Infected Bone Defects. Front. Bioeng. Biotechnol..

[48] Tan H., Ma R., Lin C., Liu Z., Tang T. (2013). Quaternized chitosan as an antimicrobial agent: Antimicrobial activity, mechanism of action and biomedical applications in orthopedics. Int. J. Mol. Sci..

[49] Xu R., Aotegen B., Zhong Z. (2017). Synthesis, characterization and biological activity of C6-Schiff bases derivatives of chitosan. Int. J. Biol. Macromol..

[50] Du X., Jiang H., Guo X., Chen L., Kang T. (2021). Synthesis of ferrocene/chitosan-AgNPs films and application in plasmonic color-switching and antimicrobial materials. React. Funct. Polym..

[51] Zhou P., Xia Z., Qi C., He M., Yu T., Shi L. (2021). Construction of chitosan/Ag nanocomposite sponges and their properties. Int. J. Biol. Macromol..

[52] Ko S.W., Lee J.Y., Rezk A.I., Park C.H., Kim C.S. (2021). In-situ cellulose-framework templates mediated monodispersed silver nanoparticles via facile UV-light photocatalytic activity for anti-microbial functionalization. Carbohydr. Polym..

[53] Alharthi S.S., Gomathi T., John Joseph J., Rakshavi J., Annie Kamala Florence J., Sudha P.N., Rajakumar G., Thiruvengadam M. (2022). Biological activities of chitosan-salicylaldehyde schiff base assisted silver nanoparticles. J. King Saud Univ. Sci..

